# Decellularized Lymph Node Scaffolding as a Carrier for Dendritic Cells to Induce Anti-Tumor Immunity

**DOI:** 10.3390/pharmaceutics11110553

**Published:** 2019-10-26

**Authors:** Hung-Jun Lin, Weu Wang, Yi-You Huang, Wei-Tsen Liao, Ting-Yu Lin, Shyr-Yi Lin, Der-Zen Liu

**Affiliations:** 1 Department of General Medicine, School of Medicine, College of Medicine, Taipei Medical University, Taipei 110, Taiwan; hrlin603@tmu.edu.tw; 2 Department of Biomedical Engineering, College of Medicine and College of Engineering, National Taiwan University, Taipei 100, Taiwan; 3 Department of Surgery, School of Medicine, College of Medicine, Taipei Medical University, Taipei 110, Taiwan; 4 Division of General Surgery, Department of Surgery, Taipei Medical University Hospital, Taipei 110, Taiwan; 5 Division of Endocrinology and Metabolism, Department of Internal Medicine, MacKay Memorial Hospital and Mackay Medical College, Taipei 104, Taiwan; 6 Department of Life Science, Tunghai University, Taichung 407, Taiwan; 7 Department of Primary Care Medicine, Taipei Medical University Hospital, Taipei 110, Taiwan; 8 TMU Research Center of Cancer Translational Medicine, Taipei Medical University, Taipei 110, Taiwan; 9 Graduate Institute of Biomedical Materials and Tissue Engineering, College of Biomedical Engineering, Taipei Medical University, Taipei 110, Taiwan; 10 Medical and Pharmaceutical Industry Technology and Development Center, New Taipei 110, Taiwan

**Keywords:** lymph node, decellularized scaffold, dendritic cell, cell-based therapy, cancer immunotherapy

## Abstract

In recent decades, the decellularized extracellular matrix (ECM) has shown potential as a promising scaffold for tissue regeneration. In this study, an organic acid decellularized lymph node (dLN) was developed as a carrier for dendritic cells (DCs) to induce antitumor immunity. The dLNs were prepared by formic acid, acetic acid, or citric acid treatment. The results showed highly efficient removal of cell debris from the lymph node and great preservation of ECM architecture and biomolecules. In addition, bone marrow dendritic cells (BMDCs) grown preferably inside the dLN displayed the maturation markers CD80, CD86, and major histocompatibility complex (MHC)-II, and they produced high levels of interleukin (IL)-1β, IL-6, and IL-12 cytokines when stimulated with ovalbumin (OVA) and CpG oligodeoxynucleotides (CPG-ODN). In an animal model, the BMDC-dLN completely rejected the E.G7-OVA tumor. Furthermore, the splenocytes from BMDC-dLN-immunized mice produced more interferon gamma, IL-4, IL-6, and IL-2, and they had a higher proliferation rate than other groups when re-stimulated with OVA. Hence, BMDC-dLN could be a promising DC-based scaffold for in vivo delivery to induce potent antitumor immunity.

## 1. Introduction

Cancer is a major cause of human death worldwide. Most cancer patients receive chemotherapy and radiation therapy, they are often only partially effective and lead to a variety of serious side effects [1]. In recent decades, cancer immunotherapy has been validated as stimulating patients’ own immune systems to eliminate cancers. Compared to chemotherapy and radiation therapy, cancer immunotherapy can specifically target tumor cells by modulating immune cells without damage to normal cells [2].

A variety of cancer immunotherapies, including DC-based cancer vaccines, chimeric antigen receptor-T lymphocyte therapies, immune checkpoint blockade antibody therapies, oncolytic vaccine, and cytokines therapies, have been developed to induce tumor-specific and cytotoxic T lymphocyte immune responses [3,4,5]. Dendritic cell based therapies have been widely investigated because dendritic cells (DCs) are thought to be the initiator in modulating immunity [6,7]. DCs are known as professional antigen-presenting cells, responsible for swallowing tumor-associated antigens, presenting them to T lymphocytes, and inducing antigen-specific immune responses. When DCs mature and become activated, they will swallow the foreign substances and subsequently present the antigenic epitopes on major histocompatibility complex (MHC) molecules [8]. At the same time, DCs up-regulate the maturation receptor, release potent cytokines [9], and migrate into lymph nodes to induce systemic immune responses.

The standard DC-based vaccine begins with first isolating DCs from the patient. Then, DCs are stimulated with the patient’s tumor-associated antigen in vitro, and the antigen-stimulated DCs are injected directly back into the patient to induce the activation of T lymphocytes [10]. The patient’s immune system will generate the tumor-specific immune response to destroy cancer cells and prevent cancer recurrence. However, several drawbacks restrict the clinical efficacy of DC therapy. First, only a few DCs actually migrate to the lymph nodes and interact with T lymphocytes, with the result of a failure to induce antitumor immune responses [11]. Moreover, the in vitro culture cannot generate sufficiently powerful DCs, with the result of a weak immune response and limited therapeutic benefit [12,13]. Thus, it is important to establish an in vitro culturing system or an in vivo DC delivery system that can more reliably induce the immune response and more potently deliver antitumor immunity.

The lymph nodes are extremely critical in responses to pathogens or danger signals, and their major functions are to filter out deleterious substances by their unique mesh microstructure, to maintain matured immunocytes, and to initiate the antigen-specific adaptive immune response [14]. The lymph nodes might lose their function because of cancer, obstruction, or infection; in such cases, surgical resection of lymph nodes is usually indicated. However, lymph node resection often causes a wide range of other problems such as increased infection, impaired cancer surveillance, and lymphedema [15]. Consequently, a promising technique for functional lymph node restoration is urgently needed.

Decellularization has emerged as a process for producing a natural extracellular matrix (ECM), which preserves the intrinsic biological cues and architectural structure of the original tissue. It provides a strategy by which to fabricate lymph node scaffolds on which to grow immune cells with immunological function. Previous studies demonstrated that spleen-derived decellularized ECM or lymph node-associated decellularized ECM can deliver immune cells and promote the organization of lymphoid-like scaffolds [16,17]; however, whether these can induce antigen-specific immunity or the antitumor immune response is still unknown. 

The aim of this study was to establish a natural decellularized lymph node scaffold (dLN), with preserved original microstructure and ECM, and to validate the ability of the dLN to support DC proliferation and maturation in vitro. The study also aimed to verify whether a bone marrow dendritic cell (BMDC)-dLN can activate a specific antitumor immune response in vivo.

## 2. Materials and Methods 

### 2.1. Decellularization of Lymph Nodes

All animal procedures were approved by the Institutional Animal Care and Use Committee (Approval No.: LAC-2015-0219, 2015-12-08). Inguinal and popliteal lymph nodes were harvested from Lewis rats. Before the decellularization process, the lymph nodes were washed in sterile, ice-cold phosphate-buffered saline (PBS) to remove blood and debris. The lymph nodes were then randomly divided into native, formic acid (FA), acetic acid (AA), and citric acid (CA) groups. The native group included fresh native lymph nodes without any treatment. For the organic acid groups, the decellularization protocols followed those used in a previous study [18]. Briefly, the lymph nodes were treated with 30% FA (pH = 1.41), 30% AA (pH = 1.98), or 30% CA (pH = 1.38), respectively. All groups were mechanically agitated in an orbital shaker at 150 rpm at 4 °C for 3 d. After each process, the dLN scaffolds were extensively washed with PBS solution in an orbital shaker at 150 rpm at 4 °C until the final pH value was stable at 7.0. The dLN scaffolds were then used as described in the following section, and all experiments were done in triplicate.

### 2.2. The Microstructure of dLN Scaffolds

The microstructure of the dLN scaffold was investigated using a scanning electron microscope (SEM). The specimens were fixed in 4% paraformaldehyde for 8 h, then washed with 0.2 M cacodylate, followed by washing with ultrapure water. The fixed specimens were dehydrated through ascending concentrations of ethanol up to pure ethanol, then dried using a critical point dryer. After being coated with gold–palladium in a mini sputter coater and mounted on metal stubs, the specimens were then observed with an SEM (Hitachi SU3500, Tokyo, Japan) at an electron-accelerating voltage of 15 kV. The pore size analysis from SEM images was performed using ImageJ software (version 1.51j8 NIH).

### 2.3. Biochemical Analysis and Histological Examination

For DNA quantification analysis, samples were dried and the weight measured. The DNA content of samples was examined using a DNA isolation kit and a Qubit™ dsDNA BR Assay Kit following the manufacturers’ instructions (Thermo Fisher Scientific, Waltham, MA, USA). In brief, samples were weighed and then digested with cell lysis buffer and proteinase K at 60 °C for 1 h. After incubation, RNase A and protein removal buffer were added, followed by centrifuging at 16,000× *g* for 3 min, to form a tight pellet. The supernatant was mixed with isopropanol and centrifuged at 16,000× *g* for 5 min. After pellet rehydration, the Qubit BR working buffer was added and measured at a wavelength of 510 nm using a Sunrise light absorbance reader (Tecan Trading AG, Männedorf, Switzerland).

To analyze the amount of glycosaminoglycans (GAGs) and total collagen, samples were digested with papain extraction reagent at 65 ℃ for 15 h. The contents of GAGs and collagen were quantified by a Blyscan Sulfated Glycosaminoglycan Assay (Biocolor) and Sirius Red Total Collagen Detection Kit (Chondrex), respectively, following the manufacturers’ instructions. The detection wavelengths of GAGs and collagen were 656 and 545 nm, respectively.

For histological examination, the tissues were fixed in a buffered 4% paraformaldehyde solution, embedded in paraffin, cut into sections 4 µm thick, and placed on silane-coated microscope slides. The sections were then stained with hematoxylin and eosin stain (HE staining) to look for the presence of cell debris, Alcian blue staining to examine glycosaminoglycans (GAGs), and Masson’s trichrome staining to detect collagen fibers.

### 2.4. Recellularization of BMDC into dLN Scaffolds

Mouse BMDCs were generated according to a previous study [19]. In brief, bone marrow cells were isolated from C57BL/6 mouse femurs and tibias and passed through 70 µm nylon meshes. The red blood cells were lysed using BD Pharm Lyse lysis buffer (BD Biosciences, San Jose, CA, USA). The remaining cells were cultured in Roswell Park Memorial Institute (RPMI) 1640 medium (containing 10% heat-inactivated fetal bovine serum (FBS), 100 U/mL penicillin, and 100 µg/mL streptomycin) supplemented with granulocyte macrophage colony-stimulating factor (1000 U/mL) and interleukin (IL)-4 (500 U/mL) at 37 °C in 5% CO_2_ for 1 week to acquire BMDC. The percentage of CD11c^+^ cells was labeled with allophycocyanin (APC) hamster antimouse CD11c monoclonal antibody (1:100) for 30 min at 4 ℃ and then examined by flow cytometry (BD FACSCanto II, BD Biosciences, San Jose, CA, USA), and the BMDCs (final percentage of CD11c^+^ cells exceeded 85%) were used for further in vitro and in vivo experiments.

BMDCs were seeded into dLN scaffolds by injection (1 × 10^6^ cells in 100 µL distributed at 3 different places), then cultured for 3 d. The recellularized lymph node scaffolds were examined for the dendritic cell marker CD11c by immunofluorescence staining of a frozen section. The samples were washed 3 times with PBS for 5 min each, then incubated with bovine serum albumin to block the nonspecific sites. Then, the samples were incubated with anti-CD11c (1:200) at 4 °C overnight. After 3 washes with PBS for 5 min each, the samples were incubated with Cy3-conjugated immunoglobulin G (1:200) and Hoechst staining for 2 h at room temperature. After washing, sections were mounted and examined with a fluorescence microscope.

### 2.5. Stimulation of BMDC-dLN and the Cytokine Profile

BMDC-dLNs were added to 100 µg/mL CPG oligodeoxynucleotides type A (CPG-ODN) (Sigma, St. Louis, Missouri, USA) and incubated with 10 or 100 µg/mL ovalbumin 257-264 (OVA) (Sigma, St. Louis, Missouri, USA) for 24 h. After incubation, the supernatants were harvested, and the concentrations of IL-1β, IL-6, and IL-12 were measured using mouse OptEIA sets according to the manufacturer’s instructions (Fisher Scientific, Waltham, MA, USA). 

To examine BMDC maturity, BMDC-dLNs were treated with 0.5% trypsin for 20 min and passed through 70 µm nylon meshes. The cells were than labeled with phyco-erythrin (PE) hamster antimouse CD80 (1:100), PE hamster antimouse CD86 (1:100), or PE hamster antimouse MHC-II monoclonal antibodies (1:100) for 30 min at 4 ℃. The expressions of CD80, CD86, or MHC-II on BMDC were measured by flow cytometry (BD FACSCanto II). The data are presented as the mean fluorescent signal for the 10,000 cells collected.

### 2.6. Immunization of Mice and Tumor Challenge

Before immunization, the dLN, BMDC, and BMDC-dLNs were pre-treated with 100 µg/mL OVA and also with 100 µg/mL CPG-ODN for 24 h. C57BL/6 mice were randomly divided into four groups (7 mice per group) and immunized with dLN, 1 × 10^6^ BMDC, 1 × 10^6^ BMDC-dLN, or PBS. All were maintained in a specific pathogen-free animal facility. For the dLN and BMDC-dLN groups, firstly, the mice were anesthetized by inhalation of isoflurane. Then, we made an incision (5 mm) with scissors above the hind paw near the abdomen, and the left inguinal lymph node was removed. The dLN or BMDC-dLN, respectively, was implanted in the same position, and the skin was closed using 6-0 nylon sutures [20]. For the BMDC-only group, the cells were given through a subcutaneous route on the left dorsal side. One week after immunization, the mice were challenged with a dorsal subcutaneous injection of 5 × 10^5^ E.G7-OVA cells (BCRC No.60418) on the right side. Tumor size was calculated twice a week for 3 weeks using the following formula:

Tumor volume (mm^3^) = length × (width^2^)/2

### 2.7. Analysis of the Cell Populations inside the BMDC-dLN after Immunization

One week after immunization, BMDC-dLNs were removed from the mice. After PBS washing, the cells were detached with 0.5% trypsin for 20 min at 37 °C, then fresh RPMI medium was added to stop the reaction. Next, samples were passed through 70 µm nylon meshes and washed with PBS 3 times. The cells were then labeled with fluorescein isothiocyanate (FITC) hamster antimouse CD3 (1:50), PE rat antimouse CD4 (1:100), and APC hamster antimouse CD11c monoclonal antibodies (1:100) for 30 min at 4 ℃. The expressions of CD3, CD4, and CD11c were measured by flow cytometry (BD FACSCanto II).

### 2.8. Ex Vivo Re-Stimulation of Splenocytes

One week after immunization, spleens were collected from mice in each group, and single-cell suspensions were prepared for ex vivo splenocyte stimulation. The spleens were passed through 70 µm nylon meshes, then incubated with BD Pharm Lyse lysis buffer for 5 min (BD Biosciences, San Jose, CA, USA). After washing with PBS, splenocytes were seeded at a density of 5 × 10^6^ cells per well in triplicate in 24-well plates and cultured in RPMI 1640 medium (containing 10% heat-inactivated FBS, 100 U/mL penicillin, and 100 µg/mL streptomycin) in the presence of 100 µg/mL OVA. After 3 d of incubation, the supernatant was collected and kept at −80 °C until use for the cytokine assay. Levels of interferon gamma (IFN-γ), IL-2, IL-4, and IL-6 were quantified using mouse IFN-γ, IL-2, IL-4, and IL-6 OptEIA sets according to the manufacturer’s instructions (Fisher Scientific, Waltham, MA, USA). 

The induction and activation of T lymphocytes were assessed by the proliferation of splenocytes in response to OVA stimulation. Splenocytes were collected by the method described above. Cells were stained with 5 µM carboxyfluorescein succinimidyl ester (CSFE) for 10 min at room temperature. After that, cells were added to prewarmed FBS and incubated for 10 min at 37 °C, then washed 3 times with PBS containing 2% FBS. CSFE-stained splenocytes were seeded at a density of 5 × 10^6^ cells/mL, then incubated with 100 µg/mL OVA for 3 d. After incubation, cells were analyzed for CSFE as FITC fluorescence intensity by flow cytometry (BD FACSCanto II).

### 2.9. Statistical Analysis

Data were reported as mean and standard deviation (SD). The significance of the data was analyzed by Mann–Whitney U tests between the native group and the decellularized groups. Statistical testing was performed using GraphPad Prism (GraphPad Software, Inc., San Diego, CA, USA), and a *p*-value < 0.01 was considered statistically significant.

## 3. Results

### 3.1. The Efficacy of the Decellularization Process

**Figure 1:**
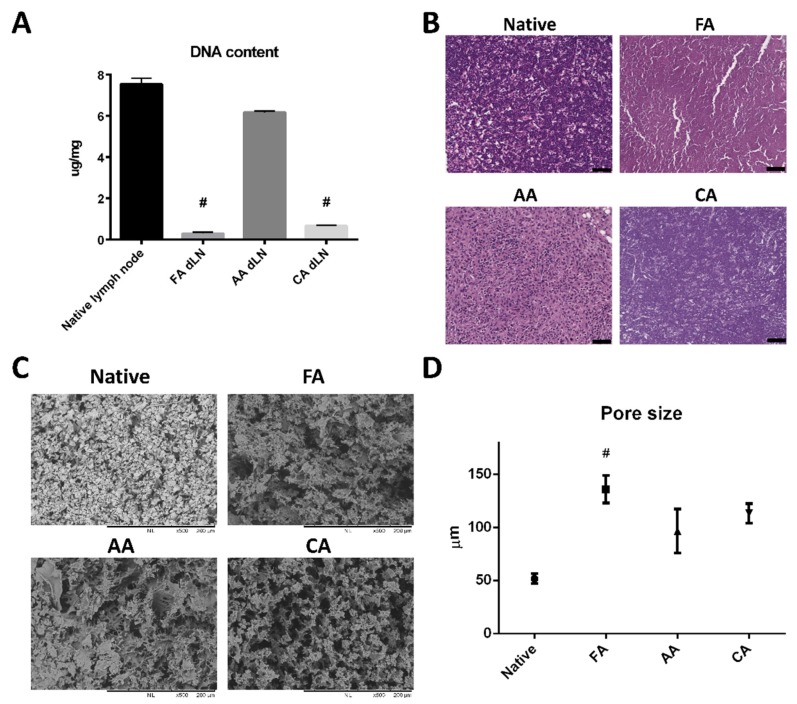
Efficacy of the organic acid decellularization process of decellularized lymph node (dLN). (**A**) Quantification of residual DNA from native lymph nodes and dLN. (Data are presented as the mean ± SD, #: *p* < 0.001.) (**B**) hematoxylin and eosin (HE) staining of lymph node scaffolds: native lymph node and lymph node treated with formic acid (FA) 30%, acetic acid (AA) 30%, and citric acid (CA) 30%. (Scale bar: 50 μm). (**C**) Microstructure of the decellularized lymph node (dLN) scaffolds. Scanning electron microscope images show the microstructures of a native lymph node and a lymph node treated with formic acid 30%, acetic acid 30%, and citric acid 30%. (**D**) Average pore sizes of the native lymph node and dLN scaffolds. (Data are presented as the mean ± SD, #: *p* < 0.001.).

The inguinal and popliteal lymph nodes were removed from rats and decellularized by FA, AA, and CA treatments. The efficiency of eliminating cellular materials was examined by a DNA qualification assay and HE staining. The results showed FA treatment had the most optimal cell removal rate (Figure 1A). The content of DNA was significantly decreased in the FA group and the CA group compared to the native lymph node group. In contrast, the AA treatment group still left a great amount of DNA in the scaffolds.

Histology examination was consistent with the results of DNA content analysis (Figure 1B). Both the native group and the AA group showed nuclear staining in the HE stain. On the other hand, the FA and CA groups had powerful decellularized images with cellular materials virtually eliminated.

### 3.2. Microstructure and ECM Examination of the dLN Scaffolds

After decellularization, the microstructure of dLN was examined by SEM. The architecture of the lymph node was well maintained after FA, AA, or CA decellularization (Figure 1C), and it showed a dense reticular meshwork. Interestingly, the average pore size of the FA dLN scaffolds (135.9 ± 12.87 μm), the AA dLN scaffold (96.49 ± 20.71 μm), and the CA dLN scaffold (113.3 ± 9.09 μm) were slightly increased in comparison to the native lymph node scaffold (51.7 ± 4.625 μm) (Figure 1D). Most importantly, the FA treatment showed the largest average pore size, which may have a beneficial effect for further BMDC recellularization.

**Figure 2:**
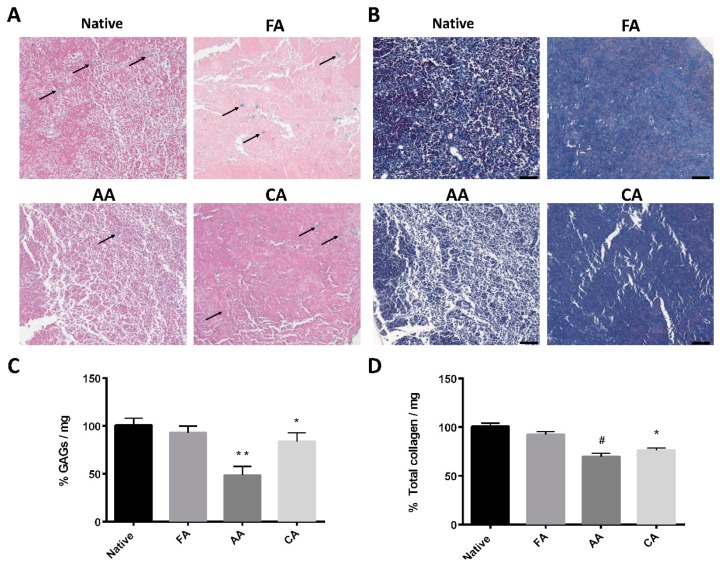
Examination of glycosaminoglycan (GAG) content (blue, black arrows) and collagen content in decellularized lymph node (dLN) scaffolds. (**A**) Alcian Blue staining of a native lymph node and lymph nodes treated with formic acid (FA) 30%, acetic acid (AA) 30%, and citric acid (CA) 30%. (Scale bar: 50 μm). (**B**) Masson’s trichrome staining of a native lymph node and lymph nodes treated with formic acid (FA) 30%, acetic acid (AA) 30%, and citric acid (CA) 30%. (Scale bar: 50 μm). Contents of (**C**) GAGs and (**D**) total collagen of lymph nodes treated with various organic acids are compared to a native lymph node. (Data are presented as the mean ± SD, *: *p* < 0.01, **: *p* < 0.05, #: *p* < 0.001.).

To investigate the GAG content after decellularization, dLN was examined with Alcian blue staining. As shown in Figure 2A, the GAG staining images after FA and CA treatment were no different than those of the native group. In contrast, GAG staining was hard to find in the AA treatment image. The amounts of GAGs significantly decreased to 48.5% ± 9.1% when lymph nodes were treated with AA, and they were slightly decreased in the CA group (84.1% ± 8.9%) (Figure 2C). On the other hand, Masson’s trichrome staining showed similar collagen preservation between the FA and native lymph node groups (Figure 2B). Figure 2D shows the preservation of total collagen before and after decellularization. Treatment with FA had no significant difference, while treatment with AA or CA reduced the total collagen content to 69.6% ± 3.4% and 76.2% ± 2.4%, respectively. These results provide useful evidence for understanding how well the FA decellularization methods preserve the lymph node ECM.

### 3.3. Maturation Marker Analysis and Cytokines Released by BMDC-dLN

**Figure 3:**
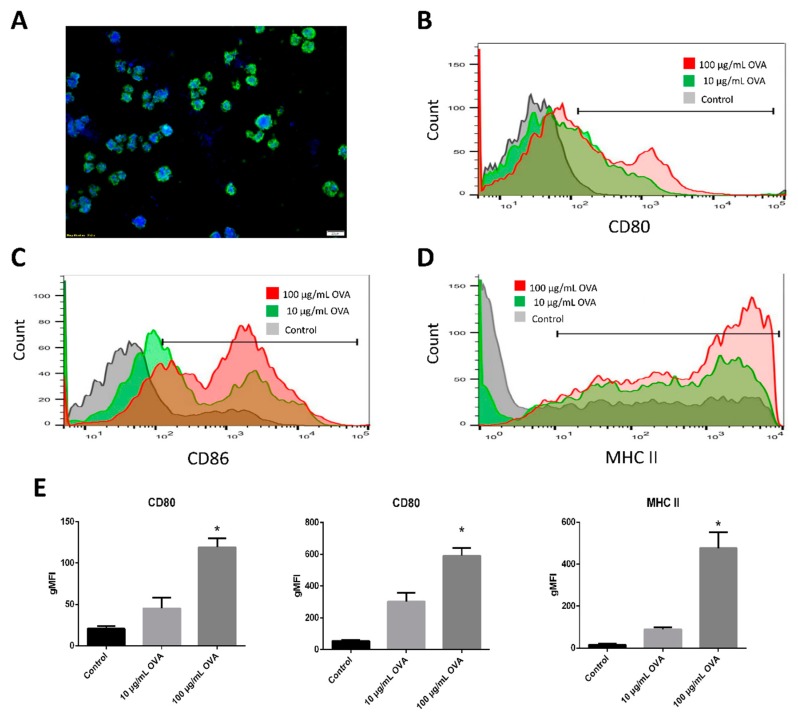
Recellularization of bone marrow dendritic cells (BMDCs) into decellularized lymph node (dLN) scaffolds. (**A**) Immunofluorescence staining with CD11c^+^ BMDC in the dLN scaffolds. BMDCs were cultured inside dLN for 24 h with 100 or 10 µg/mL OVA antigen, and 100 µg/mL CPG-ODN stimulation. Flow cytometry was used to measure the expressions of (**B**) CD80, (**C**) CD86, and (**D**) major histocompatibility complex-ІІ (MHC-ІІ). (**E**) The geometric mean fluorescence intensities (gMFIs) of CD80, CD86, and MHC-ІІ. (Data are presented as the mean ± SD, *: *p* < 0.01.).

DCs were differentiated from bone marrow stem cells, and the differentiation rate was verified by the expression of the CD11c surface marker. Over 85% of the cells presented with the CD11c^+^ marker, confirming their status as professional antigen-presenting cells, DCs. Considering the decellularization efficacy and ECM preservation, FA dLN was the optimal scaffold for the following in vitro and in vivo experiments. Next, BMDCs were injected into the dLN scaffolds and cultured for 3 d. Hoechst and anti-CD11c immunofluorescence staining revealed that the BMDC grew preferentially inside the dLN scaffolds (Figure 3A).

The co-stimulatory molecules CD80 and CD86 are key markers of DC maturation. The higher expressions of CD80 and CD86 on the surface of DCs indicate the ability of DCs to induce antigen-specific immunity. Figure 3B shows that the expression of CD80 of the BMDC-dLN was up-regulated when stimulated with OVA antigen. The CD86 presentation showed a similar elevated pattern, with CD86 expression increasing as the dose of OVA antigen increased (Figure 3C). Another key marker of activated DCs is the MHC molecule, which presents the antigen on the cell surface for T lymphocyte recognition. Flow cytometry revealed that MHC-II of BMDC was highly expressed in culture medium with the OVA antigen and CPG-ODN (Figure 3D). The results suggest that the maturation markers of BMDC could be significantly up-regulated (Figure 3E) and could have the ability to induce subsequent immune responses.

**Figure 4:**
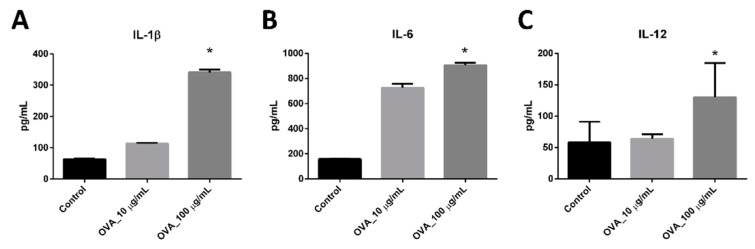
The cytokine production of bone marrow dendritic cell decellularized lymph node scaffolds (BMDC-dLN). BMDC-dLNs were treated with 10 or 100 µg/mL OVA antigen, and 100 µg/mL CPG-ODN for 24 h. The supernatants were collected, and an enzyme-linked immunosorbent assay was used to determine the production of cytokines (**A**) interleukin (IL) -1β, (**B**) IL-6, and (**C**) IL-12. (Data are presented as the mean ± SD, *: *p* < 0.01.).

The next key result was the cytokine release profile of BMDC-dLN. As shown in Figure 4A,B, BMDC-dLN stimulated with different concentrations of OVA antigen and CPG-ODN resulted in a greater production of the pro-inflammatory cytokines IL-1β and IL-6 compared to the control group. The cytokine IL-12, which is important for activation of naïve T helper lymphocytes, was also significantly released by BMDC-dLN treated with 100 µg/mL OVA (Figure 4C). These results suggest that BMDCs survived well inside the dLN and were able to mature and induce a powerful immune response when stimulated with antigen.

### 3.4. Protective Effect of the BMDC-dLN Vaccine against Tumor Challenge

**Figure 5:**
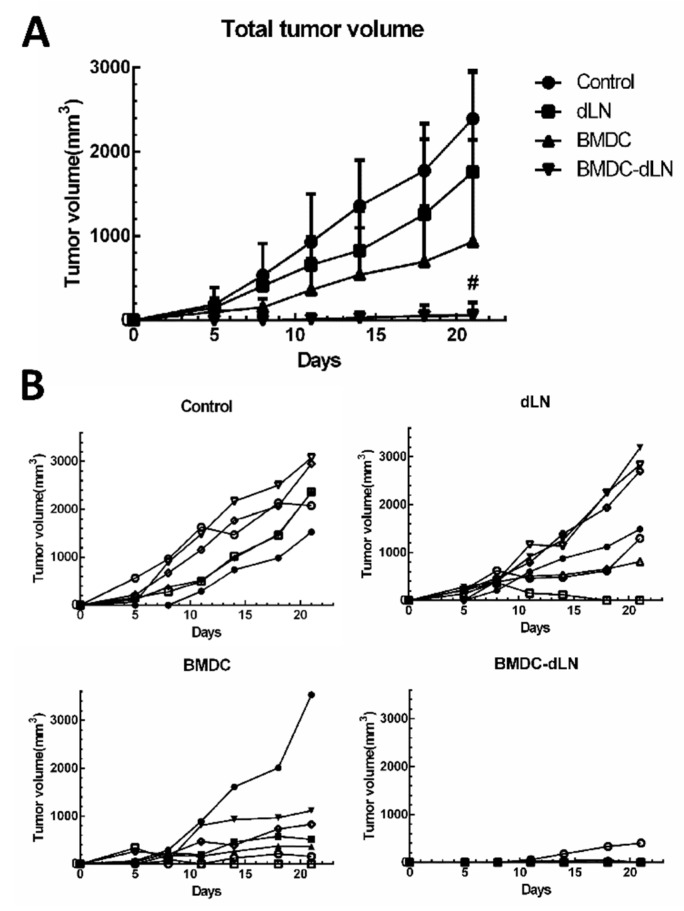
The bone marrow dendritic cell decellularized lymph node (BMDC-dLN) scaffolds rejected tumor growth in mice. (**A**) The average tumor volume of E.G7-OVA tumors in mice. (**B**) The individual tumor growth curves of E.G7-OVA tumors in mice of the phosphate-buffered saline (PBS), dLN, BMDC, and BMDC-dLN groups. (Data are presented as the mean ± SD, #: *p* < 0.001.).

To further evaluate the induction of protective immunity, BMDC-dLNs were examined against an E.G7 tumor challenge in vivo. One week before tumor inoculation, mice were immunized with BMDC-dLN implanted subcutaneously into the dorsal side. As Figure 5A shows, the mean tumor size of the dLN group was similar (1760.2 ± 1181.1 mm^3^) to the untreated control group (2392.7 ± 570.4 mm^3^). On the other hand, mice immunized with BMDC had reduced tumor growth (932.5 ± 1208.2 mm^3^). Notably, the mean tumor size of the BMDC-dLN group was significantly smaller (57.8 ± 152.9 mm^3^) than the control group, and most of the mice (6 of 7) immunized with BMDC-dLN completely rejected the E.G7-OVA tumor cells after tumor inoculation (Figure 5B). These results provide further support that the BMDC-dLN could induce an antigen-specific immune response that protects mice against a tumor challenge.

### 3.5. Analysis of Cell Populations in BMDC-dLN after Immunization

**Figure 6:**
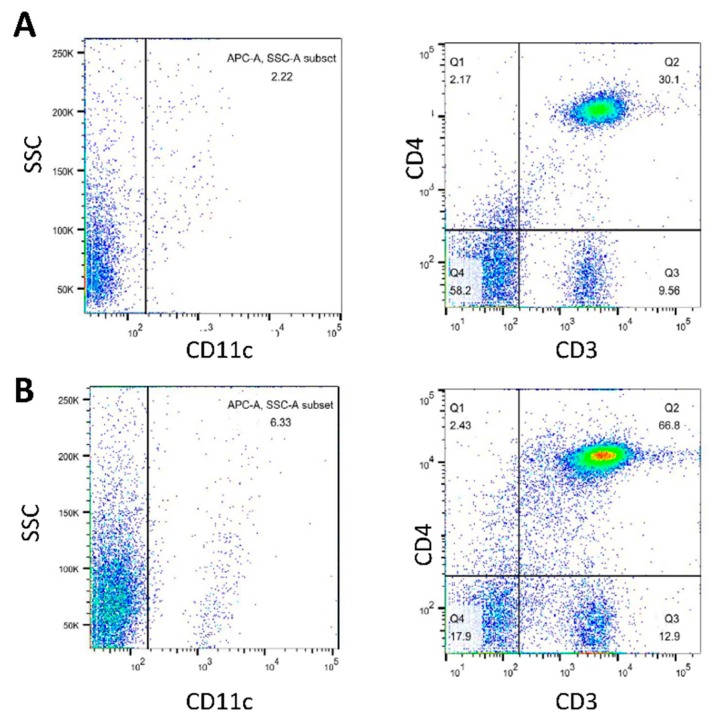
Cell populations inside the bone marrow dendritic cell decellularized lymph node scaffolds (BMDC-dLN) after in vivo immunization. After in vivo immunization, BMDC-dLNs were examined for the populations of CD3+ T lymphocytes, CD4+ T helper lymphocytes, and CD11c+ dendritic cells (DCs). (**A**) Native mouse lymph node and (**B**) BMDC-dLN.

One week after immunization, the BMDC-dLNs were removed from the mice, and the population of cells in the scaffolds was analyzed. There were 79.7% of CD3^+^ T lymphocytes and 66.8% CD3^+^CD4^+^ T helper lymphocytes in the BMDC-dLN (Figure 6). Moreover, about 6.33% of CD11c^+^ cells were observed, which demonstrated the existence of DCs. Thus, these results indicated that BMDC-dLN was a suitable environment for immune cell survival and had the potential to further induce the immune response.

### 3.6. Ex vivo Re-Stimulation of Splenocytes

**Figure 7:**
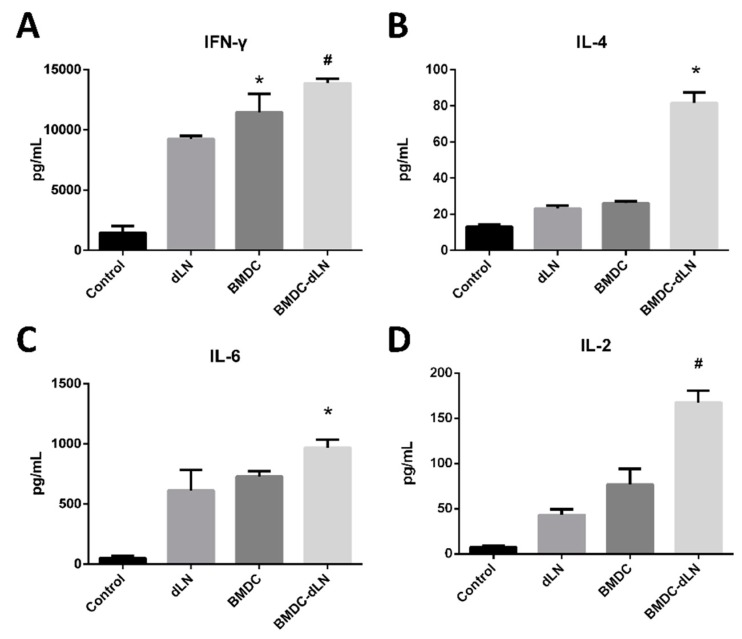
Cytokine production from splenocytes after ex vivo stimulation. Splenocytes from mice receiving different levels of immunization were obtained and cultured with 100 µg/mL OVA antigen for 3 d. The supernatants were collected, and ELISA was used to determine the production of cytokines (**A**) interferon gamma (IFN-γ), (**B**) interleukin (IL)-4, (**C**) IL-6, and (**D**) IL-2. (Data are presented as the mean ± SD, *: *p* < 0.01, #: *p* < 0.001.).

To evaluate whether splenocytes induced by BMDC-dLN were functional in vivo, splenocytes from mice were harvested 1 week after immunization and cultured with 100 µg/mL OVA for 3 d. The cytokines in the culture supernatants were analyzed to determine whether BMDC-dLNs influenced the immune response of the Th1 (IFN-γ) and Th2 (IL-4 and IL-6) pathways. The level of IFN-γ production was significantly increased in the BMDC and BMDC-dLN groups (11457 ± 1520 and 13868 ± 362.6 pg/mg, respectively), and the production of IL-6 showed similar trends as well (Figure 7). However, only the BMDC-dLN groups produced a significantly higher level of the Th2 cytokine IL-4 (81.63 ± 5.76 pg/mg) in response to OVA stimulation compared to the control group. IL-2, known as “T lymphocytes growth factor”, is produced predominantly by activated T lymphocytes. A significant increase in IL-2 secretion was found in the BMDC-dLN groups. From these data, it is apparent that BMDC-dLN has the ability to induce broader immune protection.

**Figure 8:**
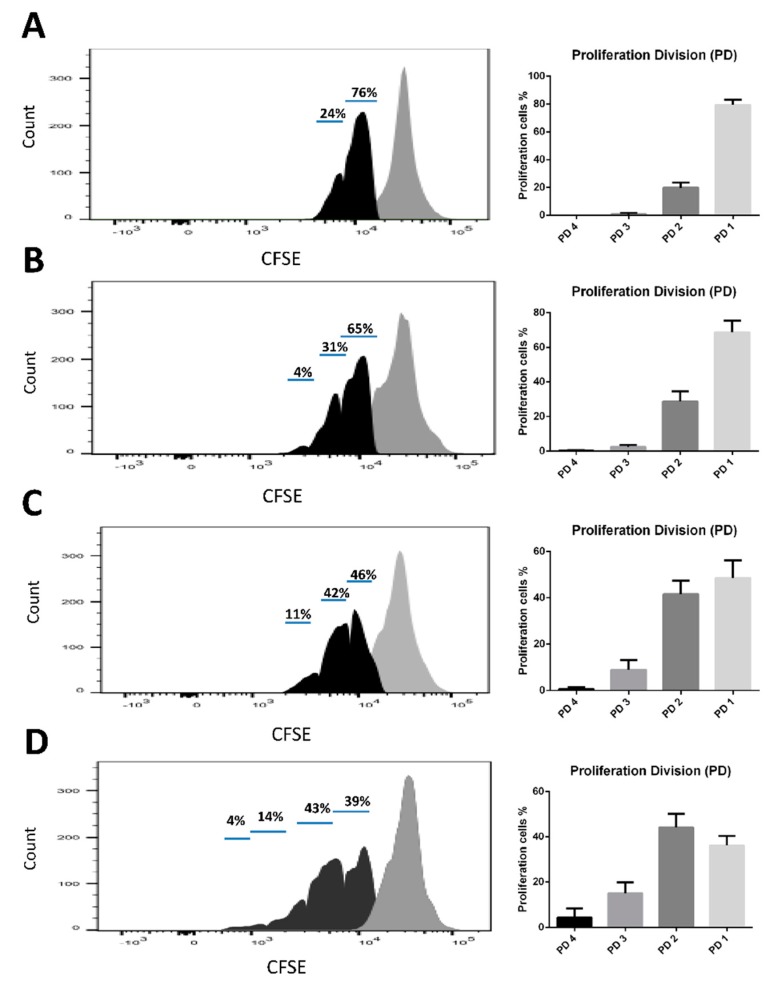
Cell proliferation of splenocytes after ex vivo stimulation. Carboxyfluorescein succinimidyl ester (CFSE)-labeled splenocytes were treated with 100 µg/mL OVA antigen for 3 d. The decrease in fluorescence intensity was examined by flow cytometry. (**A**) Phosphate-buffered saline (PBS), (**B**) decellularized lymph node (dLN), (**C**) bone marrow dendritic cell (BMDC), and (**D**) BMDC-dLN groups.

A CSFE-labeled splenocyte proliferation assay was used to verify the antigen-specific immunity of each group. The results demonstrated that the number of low CSFE fluorescence cells was increased in the BMDC-dLN group, which indicated, after 3 d of culture, that splenocytes from mice immunized with BMDC-dLN showed higher proliferation than splenocytes from BMDC- or dLN-immunized mice (Figure 8).

## 4. Discussion

Immunotherapies have emerged for cancer treatment in the past decades, but some issues still limit their clinical utility, including long processing times, lack of an ideal in vitro culture system, and unstable patient responses. Biomaterials may be able to improve cell delivery, amplify the immunomodulatory response, and, most importantly, manipulate and house immune cells in vivo [21]. In previous studies, biomaterial scaffolds have been widely used for cancer immunotherapies, including chitosan, collagen, alginate, polycaprolactone, and silica rods [22,23,24,25,26]. These materials have shown great biocompatibility as well as the ability to induce the immune response and control the release of antigens or adjuvants. Nevertheless, scaffolds with these advantages are not sufficient to keep immune cells alive, house them, and help the body maintain long-term immunity. 

Some studies have tried to fabricate a three-dimensional, lymphatic-mimicking scaffold by using hydrogel or cell-produced ECM [27,28]. Unfortunately, although the cells survived in the scaffolds, the immunological functions were still unknown. Decellularized scaffolds have the characteristics of natural tissue, such as sites for cell adhesion, the original biological cues, and a unique tissue-specific architecture [29]. The utility of decellularized scaffolds has widely focused on the tendon, bladder, blood vessels, kidney, and lung, but few on the lymph node [30,31,32]. Cuzzone et al. demonstrated that lymph nodes could be decellularized by sodium dodecyl sulfate treatment, successfully eliminating the cellular material and preserving the ECM [16]. However, other common decellularized methods, such as TRITON-X and 3-[(3-cholamidopropyl) dimethylammonio]-1-propanesulfonate, were not effective when used in lymph nodes. The reason why it is so difficult to remove all the cells is probably the dense fibrous structure of the lymph node. In addition, the study found that dLN could deliver immune cells in vivo without causing abnormal tissue inflammation. In our previous study, we demonstrated that a decellularized corneal scaffold prepared by FA treatment had near-complete cell removal and still preserved the original ECM architecture and biomolecules [18]. FA is a common decalcifying agent, and it shows great permeability through the dense structure scaffold [33]; therefore, it could be the reason that the cells in the lymph nodes were preferably eliminated by the FA decellularization treatment. AA is broadly used as cross-linker in material engineering, and it has the ability to change the porosity of ECM [34]. However, it was reported that AA was not efficient at removing residual nucleic acids from tissues [35]. Citric acid is a triprotic acid and can influence amino aldehyde bonds to damage cellular components. Nevertheless, Mathapati et al. indicated that in pericardium decellularized with citric acid, there were some negative influences on GAG content [36]. On the contrary, when lymph nodes were decellularized with FA treatment, Alcian blue staining and Masson’s trichrome staining revealed no significant loss of GAGs and collagen contents, respectively, between the native group and the dLN group. Furthermore, the quantitative tests of GAGs and total collagen also indicated that the ECM was largely preserved by FA treatment. In addition, the SEM images showed some micropores in the decellularized groups, which may facilitate the infiltration and migration of immune cells. A previous study revealed scaffolds with a pore size around 150 μm had a higher cell number than scaffolds with smaller pore sizes after seven days incubation [37]. In our study, the average pore size of the FA dLN scaffolds was 135.9 μm, which was suitable for cell attachment and growth. Taken together, the results not only showed great biocompatibility and proved that cells could survive in vitro in that environment, but they also showed the potential of this scaffolding to support the immune cells in their maturation, release of critical cytokines, and induction of antigen-specific immunity.

Although the FA-treated dLN scaffold showed perfect ECM properties, it is far more important to prove that the dLN is suitable for immune cell growth and evoking specific immunity. The presence of the markers CD80 and CD86 is critical for DC maturation, since they are responsible for the interaction with the CD28 of the T lymphocytes, resulting in cellular expansion and production of the cytokine IL-2 [38]. The results showed that BMDCs were well distributed inside the dLN, and both the maturation markers (CD80 and CD86) and the antigen-presenting marker (MHC-II) were up-regulated when stimulated with the antigen OVA and adjuvant CPG-ODN in the culture medium. 

The distinct cytokines produced by OVA-stimulated BMDC-dLNs were examined to understand the immune pathway and the strength of the immune responses. IL-1β and IL-6 act as major proinflammatory cytokines for initial immune protection [39]. IL-12, also known as T lymphocytes stimulation factor, can induce T lymphocyte activation and make T lymphocytes produce IFN-γ for enhanced cytotoxic activity [40]. The findings revealed that the level of the cytokines IL-1β, IL-6, and IL-12 were significantly increased with BMDC-dLN, indicating that the dLN preserved the optimal ECM for BMDC to survive and release potent cytokines.

The mice were subcutaneously immunized with BMDC-dLNs for one week, then the cell populations inside the BMDC-dLN were analyzed, the ex vivo splenocytes re-stimulated, and the mice given an in vivo tumor challenge. The CD3^+^ T lymphocytes, CD4^+^ helper T lymphocytes, and DCs living in the dLN were confirmed by flow cytometry, which suggested that dLNs were suitable for immunocyte housing and survival. A previous study showed adhesion and retention of immune cells inside a spleen-derived, decellularized ECM, which was consistent with our findings [17]. Moreover, in our study, splenocytes from mice immunized with the BMDC-dLN produced significantly higher levels of IFN-γ and IL-4 after ex vivo stimulation with OVA, which indicated that the BMDC-dLN could trigger better Th1 and Th2 immune responses. On the contrary, splenocytes from mice immunized with the BMDC only produced significantly higher levels of IFN-γ, which induced most of the Th1 immune response. In addition, production of the proinflammatory cytokine IL-6, which regulates DC differentiation and activation, was also up-regulated. A previous study showed that delivering cytokine IL-2 to T lymphocytes can promote more rapid T lymphocyte expansion and generation [41]. In the current study, splenocytes from BMDC-dLN-immunized mice could produce more IL-2 than other groups when re-stimulated with OVA. This result was consistent with the examination of proliferation of T lymphocytes through CSFE, with the splenocytes from the BMDC-dLN group having a higher expansion rate.

To better understand whether or not the BMDC-dLN could induce antigen-specific immunity in vivo, the E.G7-OVA tumor mouse model was evaluated. In this experiment, dLN, BMDC, and BMDC-dLN were evaluated for their ability to induce OVA-specific immunity against tumor growth in vivo. The results suggest that mice that received BMDC-dLN immunization appeared to have higher tumor-specific immunity to limit tumor growth, as shown in the six of seven mice that completely rejected the tumor challenge. In contrast, the BMDC group mostly showed only tumor growth inhibition, with just one mouse completely rejecting the tumor challenge. The results indicated that dLN successfully delivered BMDC in vivo, evoked potent cytokine production, and effectively induced strong T-cell immune responses, thus resulting in protective antitumor effects.

In accordance with these results, BMDC-dLN was shown to induce systemic cytokine production, facilitate immunocyte proliferation, and elicit potent immunoprotection. Thus, the BMDC-dLN potentially allowed the immune system to generate a coordinated immune response in vivo.

## 5. Conclusions

As cell immunotherapy for cancer treatment becomes increasingly popular, many researchers have sought to develop a promising approach for delivering immune cells. This is the first work that confirms that dLN, as a powerful scaffold to carry DCs, could induce in vitro cytokine production and in vivo antigen-specific immune responses to prevent cancer, demonstrating its relevance for further clinical application.
